# Supplementary material for: Cerivastatin Synergizes with Trametinib and Enhances Its Efficacy in the Therapy of Uveal Melanoma
Source: Cancers (Basel). 2023 Jan 31;15(3):886. doi: 10.3390/cancers15030886 (PMC9913575; doi:10.3390/cancers15030886)
Supplement: Supplementary file 1 [file cancers-15-00886-s001.zip › cancers-2139498-supplementary.pdf]

cancers

MDPI

Cerivastatin Synergizes with Trametinib and Enhances its Efficacy in the Therapy of Uveal Melanoma

Adriana Agnese Amaro, Rosaria Gangemi, Laura Emionite, Patrizio Castagnola, Gilberto Filaci, Martine J. Jager, Enrica Teresa Tanda, Francesco Spagnolo, Matteo Mascherini, Ulrich Pfeffer and Michela Croce

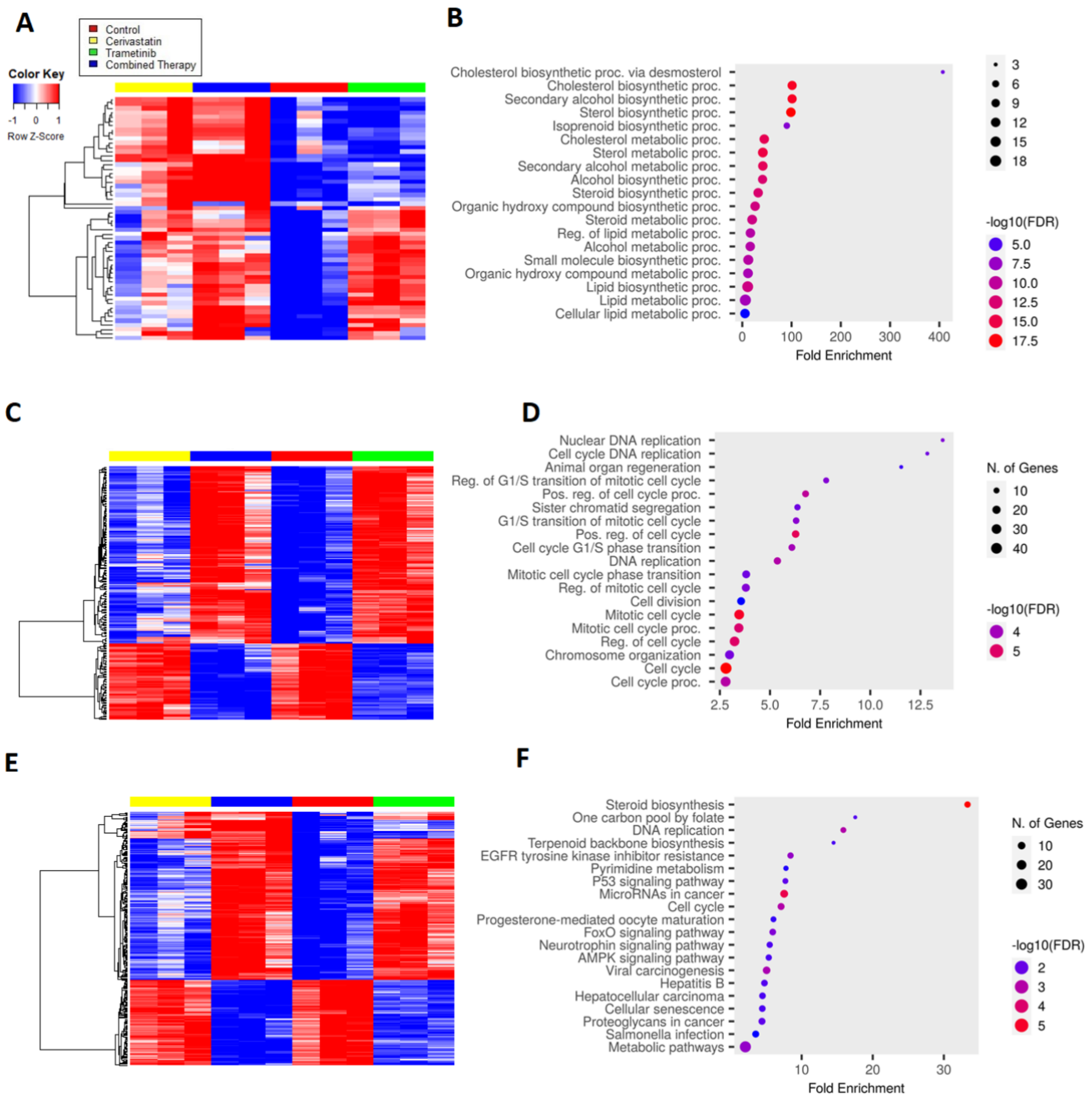

Figure S1. Gene expression profile of UPMM3 depending on treatments. The expression profiles of UPMM3 treated cells were interrogated by Significance Analysis of Microarray and the expression values of significant genes were clustered by hierarchical clustering. The expression value are reported by a colour scale (blue = expression below the mean, red = expression above the mean, black expression at the mean; the intensity is related to

the distance from the mean). The bars above the dendrogram show the treatment status (Cerivastatin treated= yellow, Untreated=red, Trametinib treated=green and combined treated =blue). A represents the differential expressed gene analysis of Cerivastatin versus untreated cells. C represents the differential expressed gene analysis of Trametinib versus untreated cells. E represents the differential expressed gene analysis of Combined drugs versus untreated cells. B, D and F represent the matched Gene set enrichment analysis for statistically significant GO biological process related to the above identified differentially expressed genes. The X-axis label represents Fold Enrichment = amount of differentially expressed genes enriched in the GO/amount of all genes in the background gene set and the Y -axis label represents GO_BP. The size and colour of the bubble represent the amount of differentially expressed genes enriched in the GO_BP and the enrichment significance (FDR calculated based on nominal P-value from the hypergeometric test.), respectively. The closer the colour is t red, the more significant the enrichment is. (ShinyGO [55] 0.76.2 http://bioinformatics.sdstate.edu/go/).

1: no treat
2: 10nM trametinib
3: 0.125μM cerivatatin
4: 10nM trametinib + 0.125 μ M cerivatatin

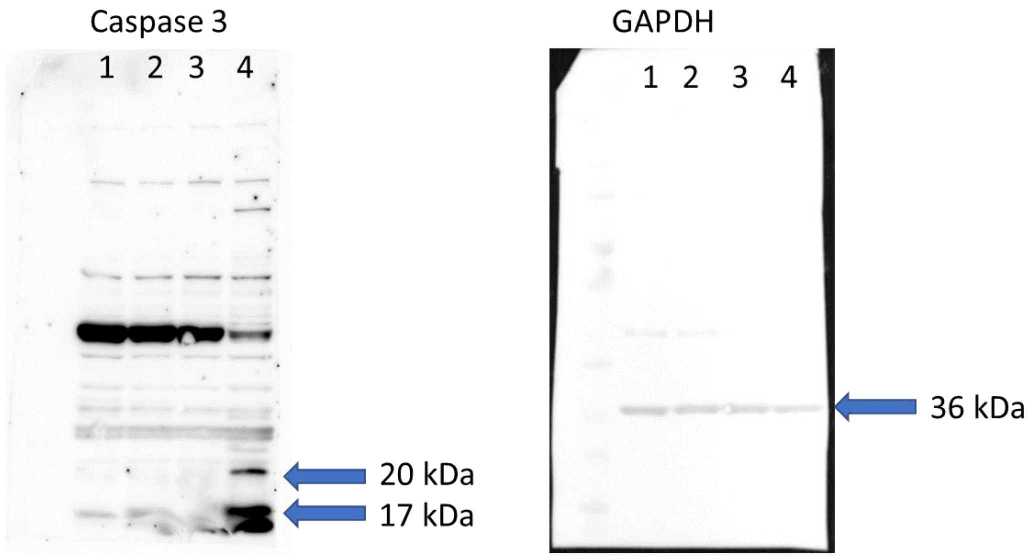

1: no treat
2: 10nM trametinib
3: 0.125 μ M cerivatatin
4: 10nM trametinib + 0.125 μ M cerivatatin

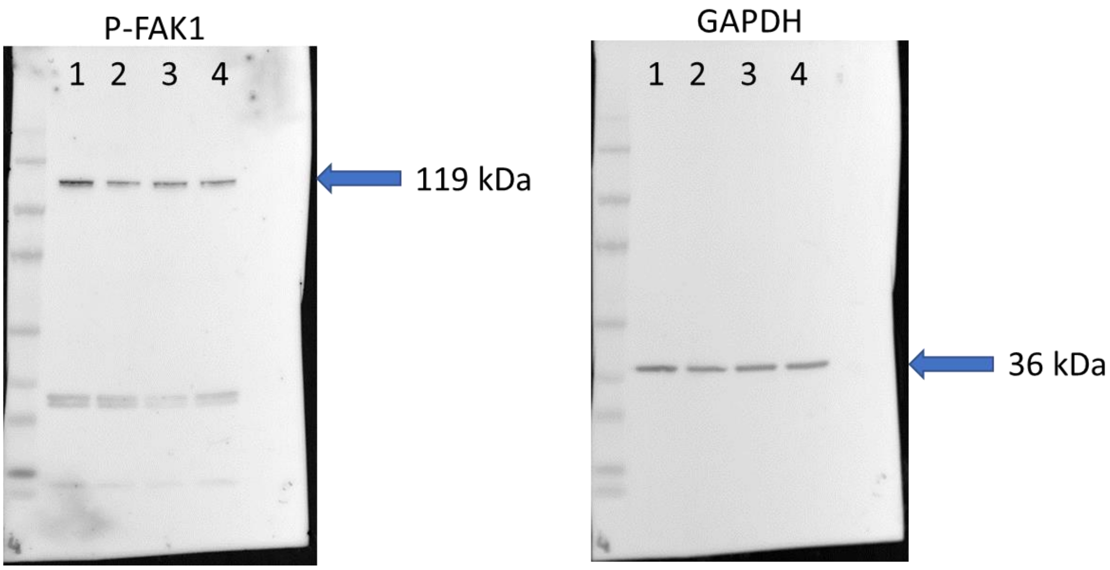

1: no treat
2: 10nM trametinib
3: 0.125 μ M cerivatatin
4: 10nM trametinib + 0.125 μ M cerivatatin

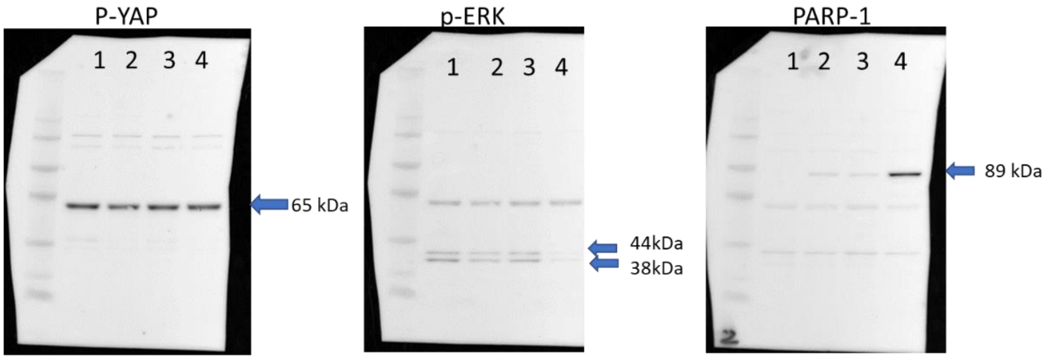

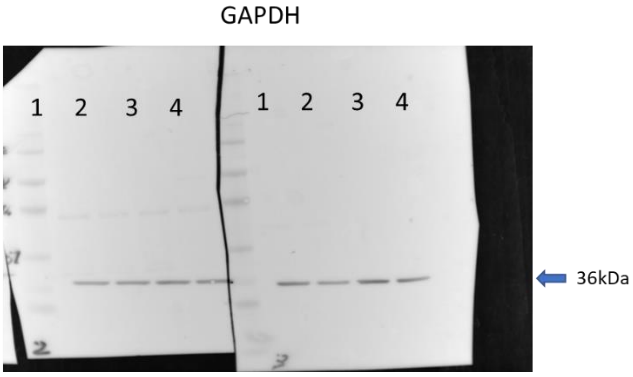

NUCLEAR EXTRACTS (panel B)

1: no treat
2: 10nM trametinib
3: 0.125 μ M cerivatatin
4: 10nM trametinib + 0.125 μ M cerivatatin

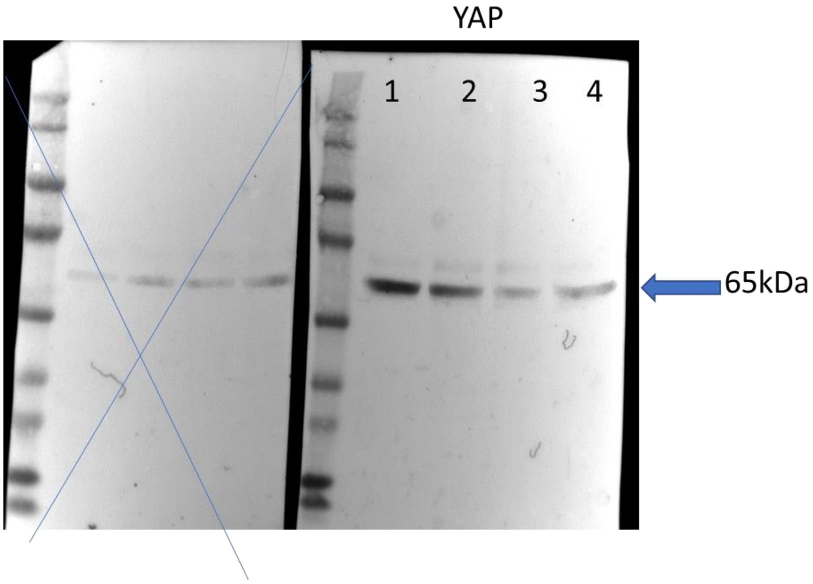

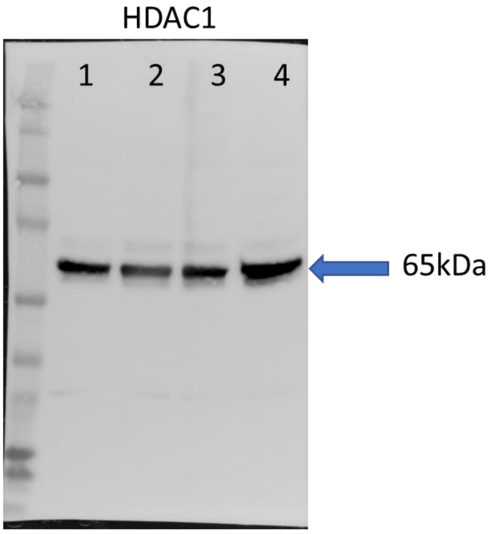

Figure S2. Original Western Blots used in this study.
